# Direct randomized evidence comparing ranibizumab and bevacizumab for macular edema secondary to retinal vein occlusion: a systematic review and meta-analysis

**DOI:** 10.1186/s12886-026-05146-4

**Published:** 2026-07-30

**Authors:** Yongfu Liang, Xiaomei Deng, Meixia Chen, Xun Zhang

**Affiliations:** 1 Ophthalmology Department, The Third Affiliated Hospital of Foshan University, Foshan, Guangdong Province, China; 2 Ophthalmology Department, Conghua District Maternal and Child Health Hospital, Guangzhou, Guangdong Province, China

**Keywords:** Retinal vein occlusion, Macular edema, Ranibizumab, Bevacizumab

## Abstract

**Background:**

Intravitreal anti–vascular endothelial growth factor therapy is the first-line treatment for macular edema secondary to retinal vein occlusion. However, direct comparative evidence between ranibizumab and bevacizumab from randomized controlled trials remains limited. This study aimed to compare the efficacy and safety of these two agents using evidence from randomized controlled trials.

**Methods:**

We systematically searched PubMed, Embase, the Cochrane Library, and Web of Science from database inception to February 11, 2026. Eligible studies were published randomized controlled trials directly comparing intravitreal ranibizumab and bevacizumab monotherapy in adults with RVO-ME. Nonrandomized and noncomparative studies were excluded.

**Results:**

Four randomized controlled trials involving 759 participants were included. At 6 months, no significant difference was observed in mean best-corrected visual acuity (BCVA) change (MD 0.61 letters, 95% CI −1.95 to 3.18). The pooled mean difference in central macular thickness (CMT) change was 1.52 μm (95% CI −237.41 to 240.46). The proportion of eyes gaining ≥ 15 ETDRS letters was comparable (RR 0.97, 95% CI 0.82 to 1.14). Safety profiles were broadly similar. Rare systemic events were infrequent and associated with considerable statistical uncertainty. Certainty of evidence was moderate for visual outcomes and low to very low for rare adverse events.

**Conclusion:**

Ranibizumab and bevacizumab show comparable short-term efficacy and safety for macular edema secondary to retinal vein occlusion, although evidence for rare systemic events remains uncertain.

**Clinical trial number:**

Not applicable.

**Trial registration:**

PROSPERO CRD420251172336.

**Supplementary Information:**

The online version contains supplementary material available at 10.1186/s12886-026-05146-4.

## Introduction

Retinal vein occlusion (RVO) is one of the most common retinal vascular disorders and a major cause of vision loss worldwide [1, 2]. It is generally classified into branch retinal vein occlusion (BRVO) and central retinal vein occlusion (CRVO) according to the site of occlusion [3]. The global prevalence of RVO is estimated to be around 0.77%, with incidence increasing markedly with age [4]. Macular edema (ME) is the most common and clinically significant complication of the disease [5] and the leading cause of vision impairment in patients with RVO [6]. Substantial proportion of patients with RVO-ME still experience poor visual outcomes despite standard treatments, highlighting the persistent therapeutic challenge [7].

Historically, laser photocoagulation, corticosteroids, and surgical interventions were used for RVO-ME [5], such as radial optic neurotomy, arteriovenous sheathotomy, and pars plana vitrectomy [8–10]. However, surgical approaches showed limited efficacy and were largely abandoned due to complications [11]. With improved understanding of RVO pathophysiology, particularly the central role of VEGF, treatment has shifted toward intravitreal therapy [5].

Before the advent of anti-VEGF therapy, laser photocoagulation was considered the standard treatment for BRVO-associated macular edema [12]. Randomized trials have since shown that adding laser to anti-VEGF therapy provides no additional visual or anatomical benefit compared with anti-VEGF monotherapy [13, 14]. Consequently, intravitreal anti-VEGF agents have become the first-line treatment for RVO-associated macular edema [15], while laser photocoagulation is now reserved for selected indications such as retinal ischemia and neovascularization [16].

Following the limited efficacy of laser therapy, corticosteroids emerged as an alternative treatment for RVO-ME. The GENEVA trial by Haller et al. provided high-level evidence supporting the dexamethasone implant (Ozurdex), demonstrating short-term visual and anatomical benefits and leading to its approval as a second-line pharmacologic option for RVO-ME after anti-VEGF therapy [15, 17]. More recently, a long-term real-world retrospective cohort study suggested that repeated dexamethasone intravitreal implant monotherapy may be associated with sustained functional and anatomical benefits and an acceptable safety profile in treatment-naive RVO-ME patients [18]. Several head-to-head randomized controlled trials comparing anti-VEGF agents with intravitreal corticosteroids showed that although early visual outcomes were comparable, anti-VEGF therapy provided superior long-term visual and anatomical outcomes and a more favorable safety profile, particularly regarding corticosteroid-related adverse events such as elevated intraocular pressure and cataract progression [19, 20]. Therefore, anti-VEGF agents have become the cornerstone of long-term management for RVO-ME due to their sustained efficacy [21].

With the establishment of anti-VEGF therapy as the mainstay of treatment for RVO-ME, several agents targeting the VEGF pathway—including ranibizumab, aflibercept, bevacizumab, faricimab, and conbercept—have been developed and clinically evaluated in both BRVO and CRVO [22–24]. For more than a decade, bevacizumab and ranibizumab have been the mainstay anti-VEGF therapies in many parts of the world [25, 26]. Notably, bevacizumab is not licensed for ocular indications but is widely administered off-label for macular edema secondary to RVO [5]. Its extensive use in clinical practice is largely driven by substantial cost differences compared with licensed anti-VEGF agents such as ranibizumab [27, 28]. Although these two drugs are often considered therapeutically interchangeable, direct head-to-head comparative evidence from randomized controlled trials is limited. Most existing comparative evidence derives from network meta-analyses or other indirect comparisons rather than from meta-analyses restricted to direct head-to-head randomized controlled trials. In particular, conclusions from network meta-analyses rely largely on indirect comparisons across heterogeneous trials and therefore may not fully reflect direct treatment effects [29–34]. Given their substantial cost difference and widespread clinical use, a rigorous comparison between ranibizumab and bevacizumab is both clinically and economically important. Direct head-to-head randomized evidence may provide a higher level of certainty than indirect comparisons by avoiding cross-trial assumptions and potential inconsistency inherent in network meta-analyses, and may also provide a more robust foundation for cost-conscious clinical decision-making in RVO-associated macular edema.

## Methods

This systematic review was performed according to the Preferred Reporting Items for Systematic Reviews and Meta-Analyses (PRISMA) 2020 Statement [35]. It was prospectively registered in the International Prospective Register of Systematic Reviews (PROSPERO; registration number: CRD420251172336).

### Search strategy

We systematically searched the PubMed, Embase, Cochrane Library, Web of Science databases to identify all relevant literature directly comparing ranibizumab and bevacizumab for the treatment of RVO-ME, with the time frame spanning from database inception to February 11, 2026.

The search strategy employed a combination of subject headings and free-text terms, including: Retinal Vein Occlusion, Macular Edema, Ranibizumab, Bevacizumab, Central Retinal Vein Occlusion, CRVO, RVO, ME, Razumab, Alymsys. Free-text terms and subject headings were connected with “OR,” while different concepts were combined using “AND.” The complete search strategy is detailed in Supplementary Table S.

In response to the discrepancy identified between the displayed and intended Boolean structure of the Embase search strategy, the Embase search was re-run on June 30, 2026 using the intended OR-based structure for the RVO abbreviation terms (“brvo”, “crvo”, and “rvo”). The updated Embase re-run search strategy is provided in Supplementary Table S1.

### Inclusion and exclusion criteria

Inclusion Criteria: 1) Randomized controlled trials directly comparing intravitreal ranibizumab and bevacizumab; 2) Adult participants (≥18 years) with macular edema secondary to branch or central retinal vein occlusion; 3) Ranibizumab and bevacizumab administered as intravitreal monotherapy; 4) Reporting extractable quantitative outcome data.

Exclusion criteria: 1) Non-randomized or quasi-randomized studies, observational designs (including cohort, case–control, and cross-sectional studies), case series, case reports, conference abstracts without full quantitative data, reviews, editorials, letters, and preclinical (animal or in vitro) studies; 2) Studies for which outcome data could not be reliably extracted after attempts to contact authors; 3) Trials involving combination therapy without extractable monotherapy arms; 4) Studies including both eyes without appropriate statistical adjustment.

### Outcomes

The primary efficacy outcomes were BCVA (ETDRS letters) and CMT (μm) at approximately 6 months (24 weeks), expressed as mean change from baseline or endpoint values.

Macular thickness measures (CMT, CST, CRT, CAT, or equivalent full-retinal central thickness parameters) were harmonized under a unified construct and analyzed as CMT when they represented mean thickness within the central macular region.

Secondary outcomes included the proportion of eyes gaining ≥ 15 ETDRS letters and ocular or systemic adverse events. Adverse events were conceptually harmonized using standardized clinical terminology to ensure consistent and conservative classification. The original reported terminology was retained in the raw data layer, and no formal adverse event coding system was applied.

The 6-month follow-up was predefined as the primary time point. When multiple time points were reported, data closest to this time window were selected. Change-from-baseline values were preferred; otherwise, endpoint values were used.

Clinically important thresholds were prespecified to aid interpretation and GRADE assessment of imprecision. A minimally important difference (MID) of 5 ETDRS letters was defined for BCVA, and 50 μm for central macular thickness. Confidence intervals entirely within these margins were considered to exclude clinically meaningful differences.

### Study selection

Two reviewers independently screened titles and abstracts of all retrieved records. Potentially eligible studies were assessed in full text according to the predefined inclusion and exclusion criteria. Particular attention was paid to confirming direct head-to-head comparison between ranibizumab and bevacizumab.

Disagreements were resolved through discussion or consultation with a third reviewer. The study selection process was documented using a PRISMA 2020 flow diagram.

### Data extraction

Data were independently extracted by two reviewers using a standardized data extraction form. The following information was collected: study characteristics (author, year, country, design, sample size), participant characteristics (age, diagnosis), intervention details (drug, dose, regimen, follow-up duration), and prespecified efficacy and safety outcomes.

For continuous outcomes, means, measures of dispersion (standard deviations, standard errors, or confidence intervals), and analyzed sample sizes at each reported time point were extracted. When time-point–specific analyzed sample sizes were not explicitly reported but analyses were stated to follow an intention-to-treat approach (e.g., with imputation methods such as LOCF), the randomized group size was used as the effective sample size.

For dichotomous outcomes, event counts and their corresponding denominators were recorded as reported. For adverse events, the safety analysis population was used when explicitly defined; otherwise, the denominator accompanying the reported event data was applied.

All outcome definitions, unit conversions, and time-point alignment rules were predefined to ensure consistency across studies.

### Risk of bias assessment

Risk of bias was assessed at the outcome level using the Cochrane Risk of Bias 2 (RoB 2) tool [36]. Five domains were evaluated (1): randomization process (2); deviations from intended interventions (3); missing outcome data (4); measurement of the outcome; and (5) selection of the reported result.

Each outcome was judged as having low risk of bias, some concerns, or high risk of bias according to the RoB 2 guidance. Assessments were conducted independently by two reviewers using predefined decision rules to ensure consistency. Disagreements were resolved through discussion.

### Statistical analysis

Meta-analyses were performed when at least two studies reported comparable outcomes. Continuous outcomes were pooled as mean differences (MDs) with 95% confidence intervals (CIs), or standardized mean differences (SMDs) when appropriate. LogMAR values were converted to ETDRS letters to ensure consistency across studies.

Dichotomous outcomes were summarized using risk ratios (RRs) with 95% CIs. A continuity correction of 0.5 was applied for single-zero studies. Double-zero studies were excluded from relative effect estimation and examined using risk difference in sensitivity analyses. For rare events, Peto odds ratios were used where appropriate, because event rates were very low and treatment effects were expected to be small and balanced.

For continuous outcomes, random-effects models were prespecified as the primary approach. For dichotomous outcomes, Mantel–Haenszel fixed-effect models were used when statistical heterogeneity was low (I^2^ < 50%), with alternative models explored in sensitivity analyses.

All analyses were conducted using R version 4.5.0 (meta and metafor packages).

### Subgroup analysis

Prespecified subgroup analyses were planned, when sufficient data were available, to explore potential sources of clinical heterogeneity. Subgroups were defined according to [1]: type of retinal vein occlusion (branch vs central); and [2] injection regimen (monthly vs pro re nata). However, they were not performed due to the limited number of eligible trials.

### Publication bias

When at least ten studies were available for a given outcome, potential publication bias was assessed by visual inspection of funnel plot symmetry and Egger’s regression test. A *p* value < 0.10 was considered suggestive of small-study effects.

When fewer than ten studies were available, formal statistical testing was not performed due to limited power.

### Certainty of evidence

The certainty of evidence for key outcomes was assessed using the Grading of Recommendations Assessment, Development and Evaluation (GRADE) framework. Evidence derived from randomized controlled trials was initially rated as high certainty and downgraded, when appropriate, based on five domains: risk of bias, inconsistency, indirectness, imprecision, and publication bias. GRADE assessments were performed using GRADEpro GDT software.

## Results

### Study selection

A total of 743 records were identified through database searching (Cochrane *n* = 51, Embase *n* = 121, PubMed *n* = 85, and Web of Science *n* = 486). After removing 9 duplicates, 734 records remained for title and abstract screening. Of these, 724 were excluded as clearly irrelevant. Ten full-text articles were assessed for eligibility. Of these, six reports were excluded for the following reasons: conference abstract without full data (*n* = 2), registry report (*n* = 2), intervention not aligned with predefined criteria (*n* = 1), and non-primary research literature (*n* = 1).

After screening the records retrieved from the updated Embase re-run, no additional eligible randomized controlled trials were identified, and the final set of included studies remained unchanged (*n* = 4) (Supplementary Figure 1.1).

**Fig. 1:**
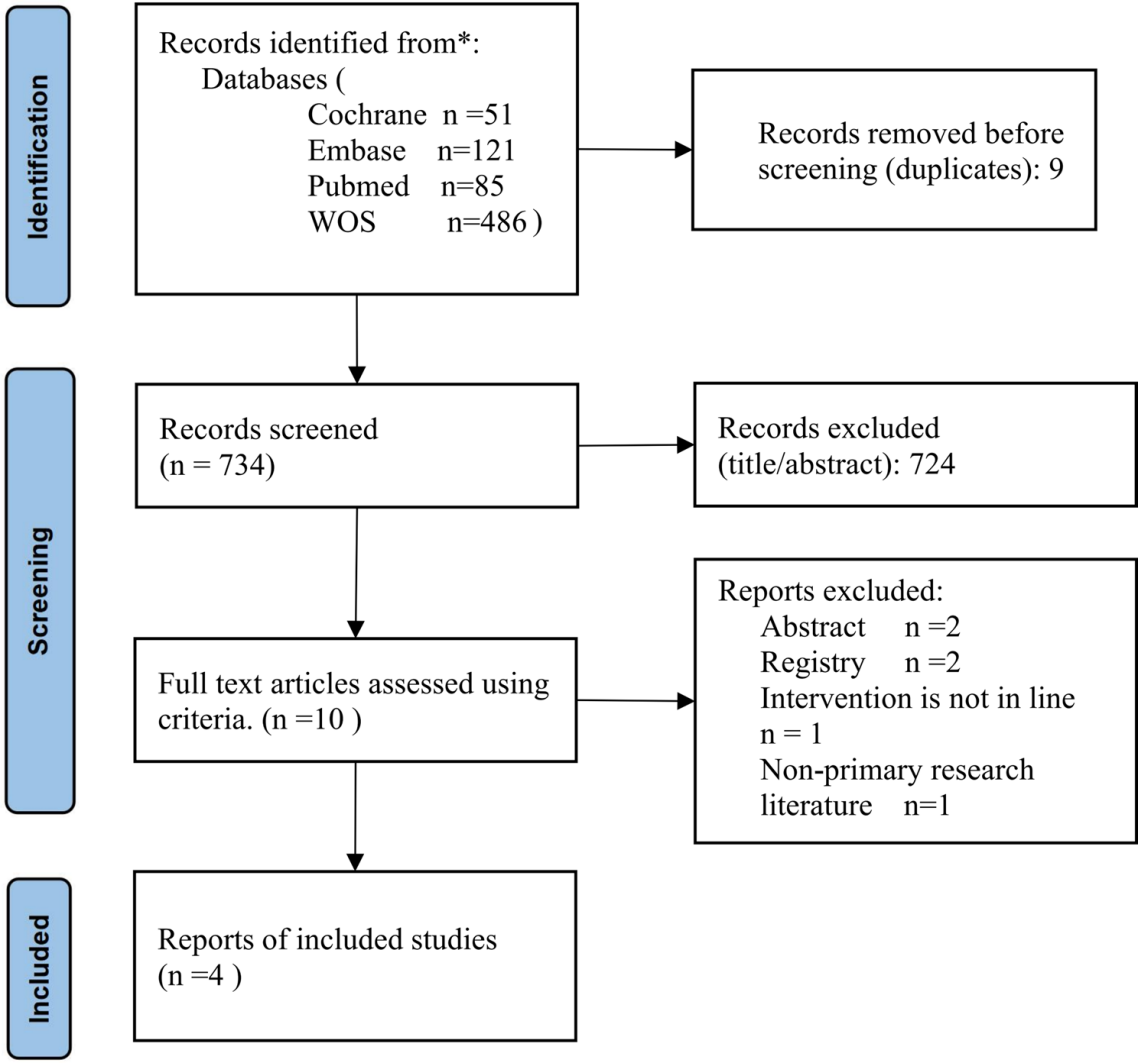
PRISMA 2020 flow diagram of study selection

Ultimately, four randomized controlled trials met the inclusion criteria and were included in the qualitative and quantitative synthesis. The study selection process is illustrated in Fig. 1, and the screening process for the updated Embase re-run is shown in Supplementary Figure 1.1.

### Study characteristics

Four randomized controlled trials published between 2015 and 2021 were included, comprising 759 participants from the ranibizumab and bevacizumab arms. Two trials enrolled mixed RVO populations, one included BRVO only, and one focused on CRVO. Sample sizes ranged from 75 to 463 participants, with mean baseline ages between 50 and 72 years.

Ranibizumab (0.5 mg) and bevacizumab (1.25 mg) were administered via intravitreal injection in all studies. Treatment strategies included fixed monthly dosing, pro re nata, and loading followed by PRN or treat-and-extend regimens. Follow-up was primarily 6 months, except for one study reporting outcomes up to 100 weeks. Detailed characteristics are summarized in Table 1, and baseline clinical and phenotypic characteristics are further summarized in Supplementary Table 1A. Across the included trials, baseline visual acuity and central macular thickness were generally comparable between ranibizumab and bevacizumab arms within each study. However, clinical phenotypes varied across studies, including differences in RVO subtype, disease duration, prior treatment status, baseline macular thickness, and the extent to which macular ischemia or other ischemia/perfusion-related information was reported or incorporated into eligibility criteria.

**Table 1:** Detailed characteristics of literature

Study_ID	Country	Study_design	Sample_size	RVO Type	Mean Age Ranibizumab/Bevacizumab (years)	BCVA Ran/Bev(Mean±SD, ETDRS letters/LogMAR)	OCT-Macular Ran/Bev thickness (Mean±SD, μm)	Ran	Bev	Administration	Injection_regimen	Follow-up	Reported outcomes
Narayanan 2015	India	RCT	75 (Ranibizumab:37, Bevacizumab 38)	BRVO	53/50	52.81 ± 14.41/ 56.1 ± 10.01	445.65 ± 119.53/ 491.55 ± 155.09	0.5 mg	1.25 mg	Intravitreal injection	PRN after baseline	6 months (monthly visits)	Change in BCVA at 6 months; Change in CRT at 6 months; Proportion gaining ≥ 15 ETDRS letters; Safety outcomes
Rajagopal 2015	United States	RCT	98 (Ranibizumab:49, Bevacizumab 49)	Mixed RVO	72.4/70.6	0.73 ± 0.45/ 0.76 ± 0.38	517.2 ± 159.8/ 538.0 ± 211.0	0.5 mg	1.25 mg	Intravitreal injection	Fixed monthly injections for 6 months	12 months (monthly visits)	Change in central foveal thickness at 6 months; Change in BCVA at 6 months; Proportion achieving CFT < 275 µm; Proportion achieving fluid-free maculae; Proportion gaining ≥ 0.3 logMAR; Proportion losing ≥ 0.2 logMAR; Safety outcomes
Vader 2020	Netherlands	RCT	277 (Ranibizumab 138 Bevacizumab 139)	Mixed RVO	67.4/68.3	59 ± 16.7/ 60.3 ± 14.8	615.2 ± 217.3/ 602.3 ± 201.2	0.5 mg	1.25 mg	Intravitreal injection	Fixed monthly injections for 6 months	6 months (monthly visits)	Change in BCVA from baseline at 6 months (primary); Change in central area thickness; Proportion gaining ≥ 15 letters; Safety outcomes
Hykin 2021	UK	RCT	463 (Ranibizumab:155, Bevacizumab 154, Aflibercept 154)	CRVO	69.2/69.3	53.6 ± 15.1/ 54.4 ± 14.2	731.3 ± 227.6/ 676.1 ± 207.0	0.5 mg	1.25 mg	Intravitreal injection	Loading phase (3 monthly injections) followed by PRN/Treat-and-Extend	100 weeks (visits at 12, 24, 52, 76 and 100 weeks)	Change in BCVA letter score from baseline to 100 weeks (primary); visual acuity categorical outcomes; OCT outcomes; number of injections; safety outcomes

RVO = Retinal Vein Occlusion; BRVO = Branch Retinal Vein Occlusion; CRVO = Central Retinal Vein Occlusion; RCT = Randomized Controlled Trial; PRN = Pro Re Nata; BCVA = Best-Corrected Visual Acuity; OCT = Optical Coherence Tomography; CRT = Central Retinal Thickness; CST = Central Subfield Thickness; CMT = Central Macular Thickness; Ran = Ranibizumab; Bev = Bevacizumab;

### Risk of bias

**Fig. 2:**
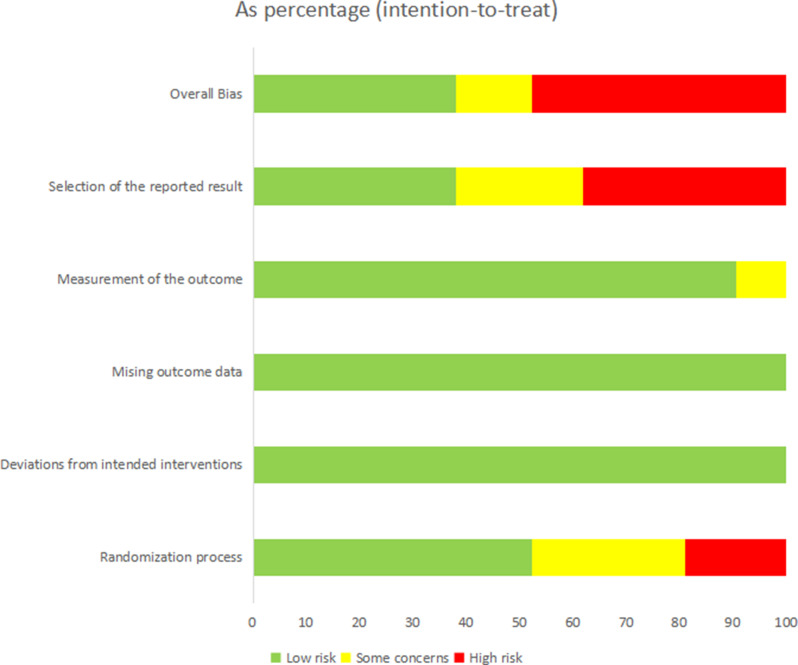
RoB2 domain-level summary bar plot

Risk of bias was assessed at the outcome level using the Cochrane Risk of Bias 2 (RoB 2) tool (Table 2; Fig. 2).

**Table 2:** Outcome level risk-of-bias judgment

Study ID	Outcome	D1	D2	D3	D4	D5	Overall
Narayanan 2015	BCVA Mean change from baseline at 6 month						
Rajagopal 2015	BCVA Mean change from baseline at 6 month						
Vader 2020	BCVA Mean change from baseline at 6 month						
Narayanan 2015	Hypertension any time						
Vader 2020	Hypertension any time						
Narayanan 2015	Intraocular pressure increased any time						
Vader 2020	Intraocular pressure increased any time						
Hykin 2021	Intraocular pressure increased any time						
Rajagopal 2015	Myocardial infarction any time						
Vader 2020	Myocardial infarction any time						
Hykin 2021	Myocardial infarction any time						
Narayanan 2015	≥15-letter gain Month 6						
Rajagopal 2015	≥15-letter gain Month 6						
Vader 2020	≥15-letter gain Month 6						
Narayanan 2015	macular thickness Mean change from baseline at 6 month						
Vader 2020	macular thickness Mean change from baseline at 6 month						
Narayanan 2015	Retinal neovascularisation any time						
Hykin 2021	Retinal neovascularisation any time						
Rajagopal 2015	Stroke any time						
Vader 2020	Stroke any time						
Hykin 2021	Stroke any time

Across outcomes, concerns were most frequently identified in Domain 5 (selection of the reported result), particularly for efficacy outcomes that allowed multiple eligible measurements or analytic approaches. For these outcomes, several studies were judged to be at high risk of bias due to concerns regarding the availability of prespecified analysis plans and the potential for selective reporting.

In contrast, Domains 2 (deviations from intended interventions), 3 (missing outcome data), and 4 (measurement of the outcome) were predominantly assessed as low risk across studies and outcomes. Safety outcomes, including intraocular pressure increase, myocardial infarction, stroke, and retinal neovascularisation, were generally judged to be at low risk of bias.

For the primary efficacy outcomes, two studies were judged to be at high risk of bias, mainly driven by concerns in the randomization process (Domain 1) and selection of the reported result (Domain 5). One study was consistently judged to be at low risk of bias across most assessed outcomes.

### Primary outcomes

#### BCVA - mean change from baseline at 6 months

Three randomized controlled trials (Narayanan 2015, Rajagopal 2015, Vader 2020) reported mean change in BCVA at approximately 6 months and were included in the meta-analysis.

**Fig. 3:**
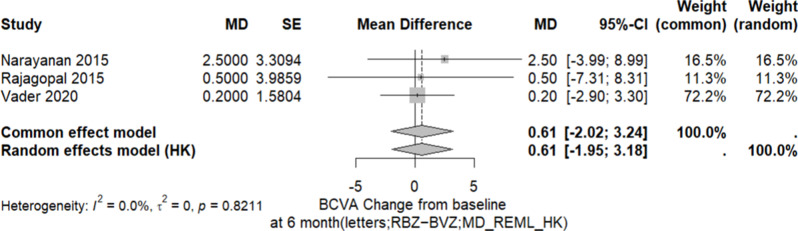
Forest plot of BCVA change at 6 months

Main analysis (random-effects model, REML + Hartung–Knapp), MD 0.61 letters (95% CI −1.95 to 3.18). There was no statistically significant difference in BCVA improvement between intravitreal ranibizumab and bevacizumab at 6 months. No statistical heterogeneity was observed (I^2^ = 0%; τ^2^ = 0; *p* = 0.821). The common-effect model yielded a nearly identical estimate MD 0.61 letters (95% CI −2.02 to 3.24) (Fig. 3).

**Fig. 4:**
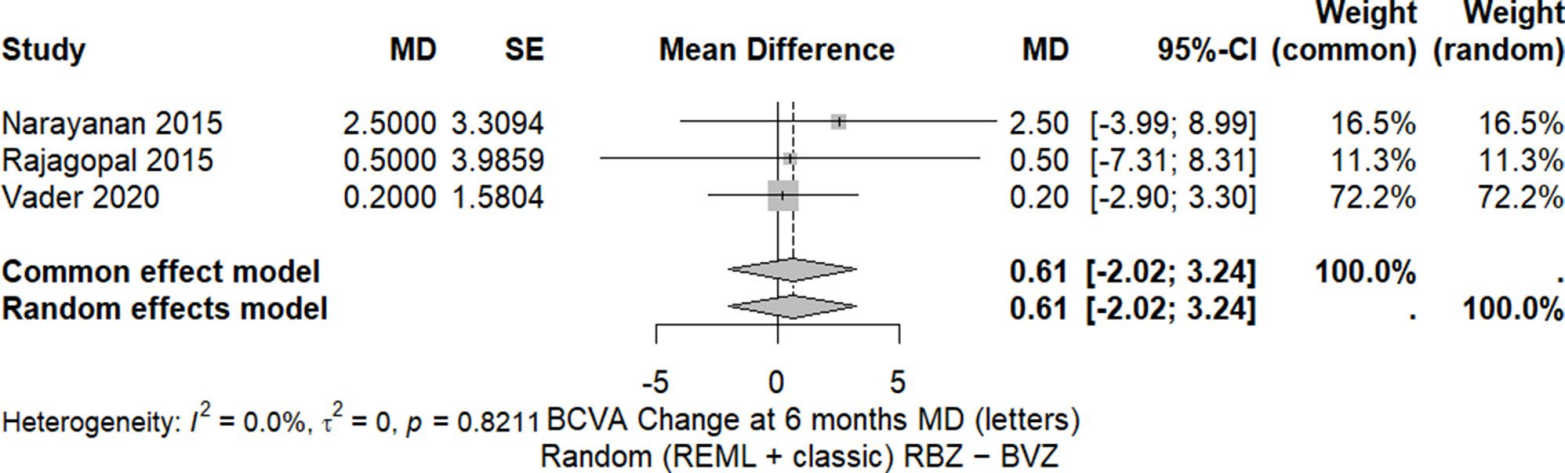
Sensitivity analysis Forest plot of BCVA change at 6 months REML + classic

**Fig. 5:**
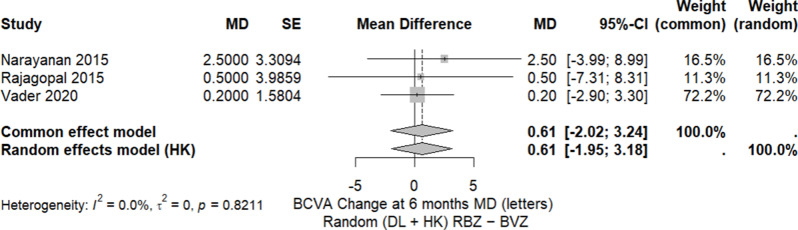
Sensitivity analysis Forest plot of BCVA change at 6 months DL + HK

We performed one-at-a-time sensitivity analyses by holding the effect estimates and data inputs constant while varying key modeling components. Sensitivity analyses using alternative random-effects specifications showed consistent results: REML + classic MD 0.61 (95% CI −2.02 to 3.24) (Fig. 4). DL + HK: MD 0.61 (95% CI −1.95 to 3.18) (Fig. 5).

At 6 months, ranibizumab and bevacizumab resulted in comparable improvements in BCVA, with no evidence of a clinically important difference between treatments.

**Fig. 6:**
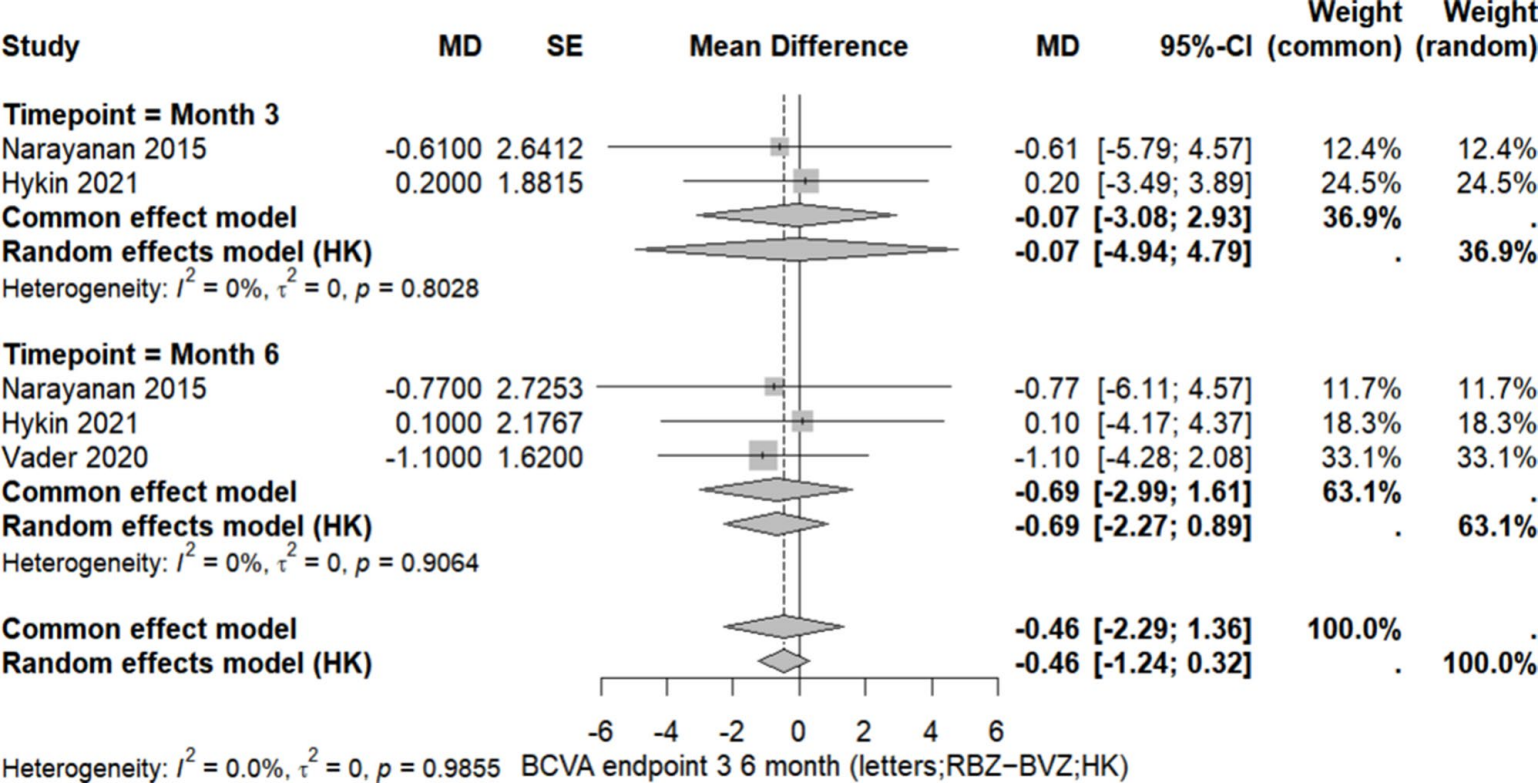
Forest plot of BCVA endpoint at 3 and 6 months (subgroup by time point)

In addition to the primary analysis based on change-from-baseline BCVA at 6 months, endpoint analyses at 3 and 6 months were conducted as supportive assessments. These analyses yielded effect estimates close to the null, consistent with the primary findings, and demonstrated no evidence of a treatment difference across time points and outcome definitions (Fig. 6).

#### OCT-based macular thickness – mean change from baseline at 6 months

Two randomized controlled trials (Narayanan 2015 and Vader 2020) reported mean change in central macular thickness at 6 months.

**Fig. 7:**
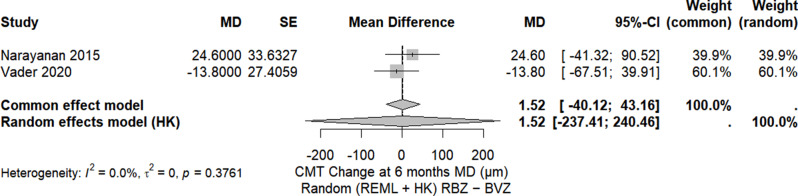
Forest plot of CMT change at 6 months

Main analysis (random-effects model, REML + Hartung–Knapp), MD 1.52 μm (95% CI −237.41 to 240.46). The confidence interval was wide and crossed the line of no effect. Statistical heterogeneity was not detected (I^2^ = 0%; τ^2^ = 0; *p* = 0.38). The common-effect model yielded MD 1.52 μm (95% CI −40.12 to 43.16) (Fig. 7).

**Fig. 8:**
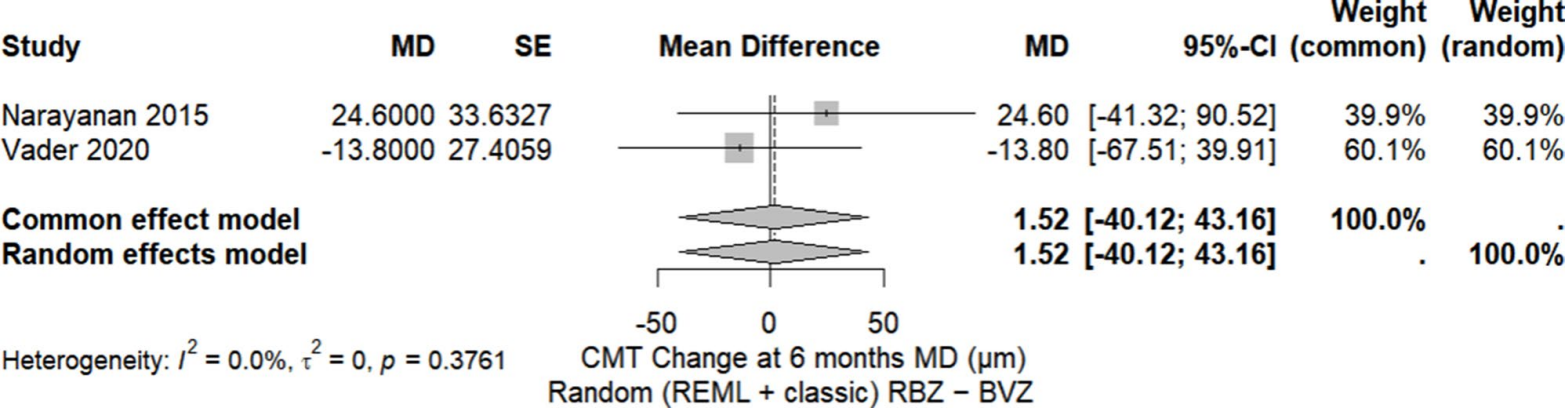
Sensitivity analysis Forest plot of CMT REML + classic

**Fig. 9:**
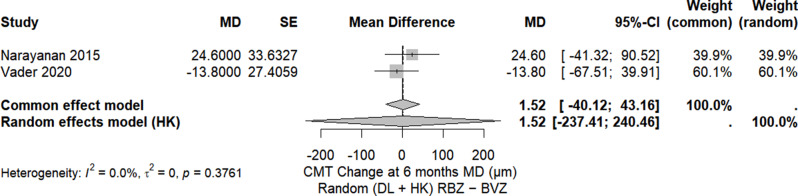
Sensitivity analysis Forest plot of CMT DL+HK

Sensitivity analyses using a random-effects model with classic confidence intervals (REML + classic) produced MD 1.52 μm (95% CI −40.12 to 43.16) (Fig. 8). Similarly, applying the DerSimonian–Laird estimator with Hartung–Knapp adjustment resulted in MD 1.52 μm (95% CI −237.41 to 240.46) (Fig. 9).

The prespecified random-effects model with Hartung–Knapp adjustment yielded a very wide 95% confidence interval (−237.41 to 240.46 μm), reflecting substantial statistical uncertainty due to the small number of included studies (k = 2). Although the point estimate was close to zero, the interval crossed the prespecified minimally important difference (±50 μm), indicating that clinically meaningful differences cannot be excluded.

Notably, with only two studies, the Hartung–Knapp method relies on a t-distribution with one degree of freedom (df = 1), resulting in a large critical value and consequently wide confidence intervals. This expansion is attributable to the inherent small-sample adjustment of the method rather than to between-study heterogeneity, as the estimated between-study variance (τ^2^) was 0 under both REML and DerSimonian–Laird estimators.

For reference, the common-effect model produced a narrower confidence interval within the ±50 μm margin; however, this finding should be interpreted cautiously given the limited number of trials and the restricted information base.

### Secondary outcomes

Three randomized controlled trials (Narayanan 2015, Rajagopal 2015, Vader 2020), including 450 participants, reported the proportion of patients achieving a gain of at least 15 ETDRS letters at 6 months.

**Fig. 10:**
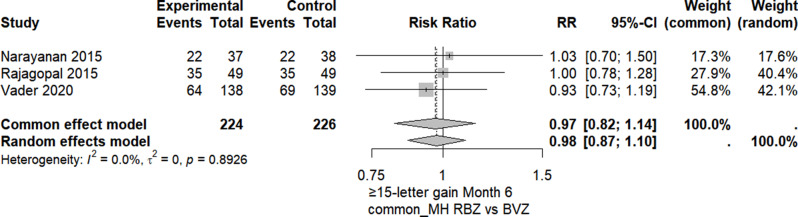
Forest plot of ≥15-letter gain at 6 months

Main analysis (common-effect model, Mantel–Haenszel), There was no statistically significant difference between ranibizumab and bevacizumab with RR 0.97 (95% CI 0.82 to 1.14). The confidence interval crossed the line of no effect (RR = 1). Statistical heterogeneity was not detected (I^2^ = 0%; τ^2^ = 0; *p* = 0.89). The random-effects model yielded a similar estimate RR 0.98 (95% CI 0.87 to 1.10). Results were consistent across models (Fig. 10).

At 6 months, ranibizumab and bevacizumab resulted in similar proportions of patients achieving a ≥ 15-letter visual gain.

### Safety outcomes

Adverse events were infrequently reported across trials and are summarized in Table 3.

**Table 3:** Safety outcomes

Outcome	Studies (n)	Model	Effect estimate (95% CI)	I^2^	Ranibizumab events/total	Bevacizumab events/total
Hypertension	2	Mantel–Haenszel (fixed effect)	RR 0.97 [0.59; 1.58]	0.00%	26/177	27/178
Intraocular pressure increased	3	Mantel–Haenszel (fixed effect)_0.5(only 0)	RR 1.82 [0.80; 4.14]	0.00%	15/332	8/332
Myocardial infarction	3	Peto (fixed effect)	OR 0.13[0.02; 0.95]	0.00%	0/344	4/343
Retinal neovascularisation	2	Mantel–Haenszel (fixed effect)	RR 1.52 [0.26; 8.88]	0.00%	3/192	2/192
Stroke	3	Peto (fixed effect)	OR 1.94[0.20; 18.72]	62.50%	2/344	1/343

No clear differences between ranibizumab and bevacizumab for hypertension (RR 0.97, 95% CI 0.59 to 1.58), intraocular pressure increase (RR 1.82, 95% CI 0.80 to 4.14), or retinal neovascularisation (RR 1.52, 95% CI 0.26 to 8.88).

Myocardial infarction was rare (four events in total, all occurring in the bevacizumab group). Using the prespecified Peto fixed-effect method, the pooled odds ratio suggested a potential signal favoring ranibizumab (OR 0.13, 95% CI 0.02 to 0.95). However, given the extremely sparse data and the sensitivity of the estimate to alternative analytical approaches, this finding should be interpreted cautiously. Sensitivity analyses using Mantel–Haenszel models with continuity correction and risk-difference models were not statistically significant, and the absolute risk difference was small.

Stroke events were also rare (three events in total), and individual trials suggested opposing directions of effect. The Peto fixed-effect model yielded an OR of 1.94 (95% CI 0.20 to 18.72), with moderate heterogeneity (I^2^ = 62.5%). Sensitivity analyses using Mantel–Haenszel models with alternative continuity-correction strategies produced similar, non-significant results, and the pooled absolute risk difference was close to zero RD 0.00 (95% CI −0.01 to 0.02). Overall, the extremely sparse data preclude reliable comparative inference.

Across safety outcomes, sensitivity analyses using alternative statistical models, effect measures, and continuity-correction strategies did not materially alter the overall conclusions. Forest plots for all safety outcomes and their corresponding sensitivity analyses are provided in the Supplementary Material (Supplementary Figures S1–S5).

### Publication bias

As fewer than ten studies were included for each outcome, formal assessment of publication bias (e.g., funnel plot asymmetry or Egger’s test) was not performed due to limited statistical power.

### Certainty of evidence

**Fig. 11:**
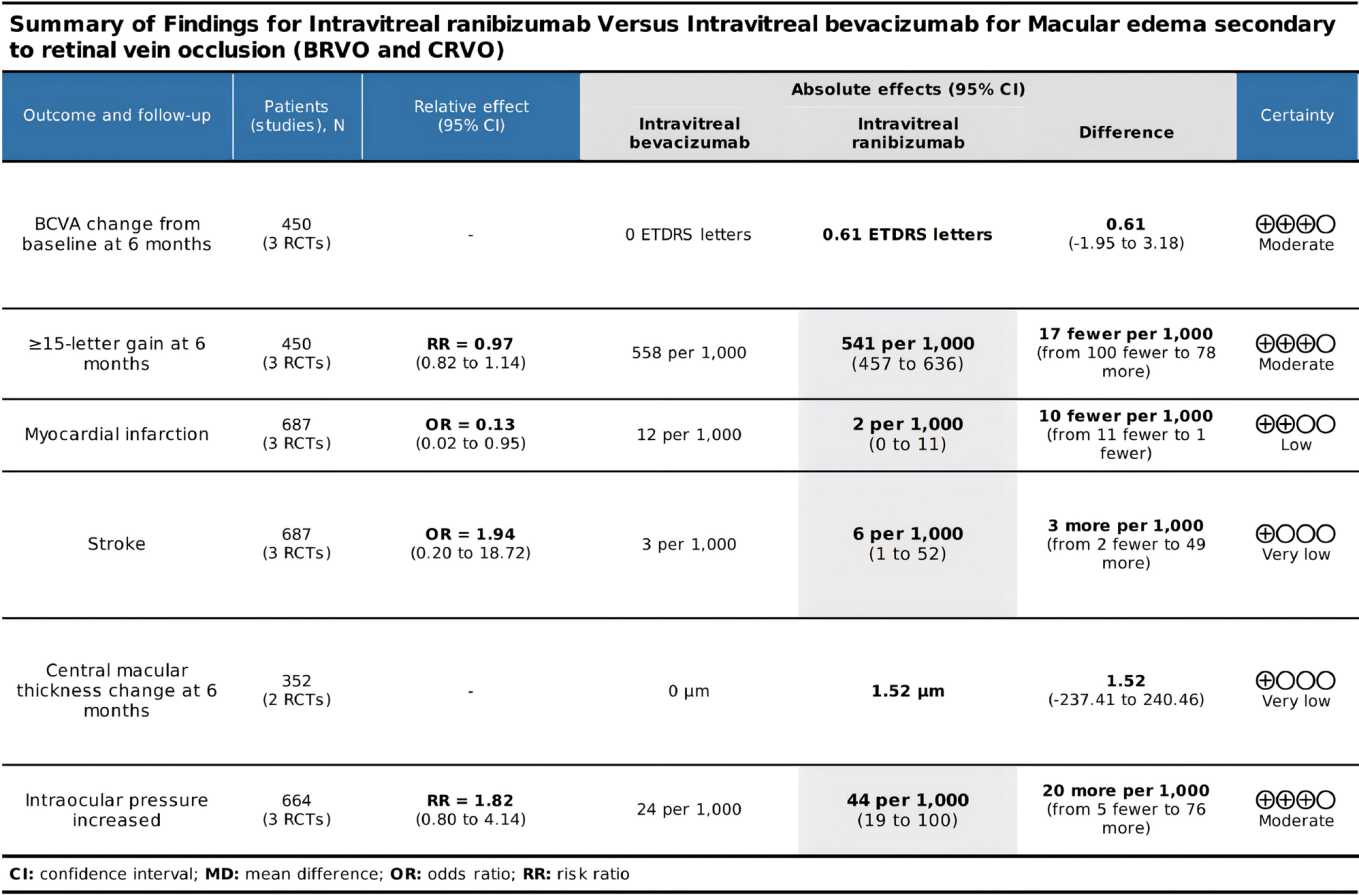
Summary of findings

The certainty of evidence was assessed using the GRADE approach and is summarized in the Summary of Findings (Fig. 11).

For critical efficacy outcomes at 6 months, the certainty of evidence was rated as moderate. The estimate for BCVA change was downgraded one level for risk of bias due to concerns regarding selective reporting, while inconsistency and imprecision were not considered serious. The confidence interval excluded the prespecified minimally important difference (±5 ETDRS letters), indicating no clinically meaningful difference between treatments. Similarly, the certainty of evidence for the proportion of participants gaining ≥ 15 letters was rated as moderate, downgraded one level for risk of bias.

For safety outcomes, certainty varied according to event frequency. The certainty of evidence for intraocular pressure increase was moderate, downgraded one level for imprecision. In contrast, the certainty of evidence for myocardial infarction was low, downgraded two levels for very serious imprecision due to sparse events and wide confidence intervals. The certainty of evidence for stroke was very low, downgraded for inconsistency and very serious imprecision.

For central macular thickness change at 6 months, the certainty of evidence was rated as very low, downgraded for risk of bias and very serious imprecision due to an extremely wide confidence interval crossing both the null effect and the prespecified clinically important difference (±50 μm).

Overall, the certainty of evidence across critical visual outcomes was moderate, while certainty for rare systemic adverse events was low or very low due to sparse data.

## Discussion

In this systematic review and meta-analysis of randomized controlled trials directly comparing intravitreal ranibizumab and bevacizumab for macular edema secondary to retinal vein occlusion, pooled estimates did not demonstrate a statistically significant difference between the two agents at 6 months. Importantly, this finding was consistent across analytical approaches, outcome definitions, and time points, with similar results observed in both change-from-baseline and endpoint analyses at 3 and 6 months, supporting the robustness of the evidence. Consistent patterns were also observed across anatomical and categorical efficacy outcomes, as well as safety endpoints. Overall, current randomized evidence does not demonstrate superiority of either agent for short-term efficacy outcomes.

Although BCVA is a patient-relevant and widely used functional endpoint in RVO-ME trials, visual acuity may be influenced by factors beyond macular edema resolution, including macular ischemia, disease chronicity, baseline visual impairment, and irreversible retinal structural damage. Therefore, BCVA-based findings should be interpreted together with OCT-based anatomical outcomes and categorical visual response measures. In the present analysis, central macular thickness was evaluated as a complementary anatomical endpoint, and the proportion of patients gaining ≥ 15 ETDRS letters was analyzed as an additional clinically interpretable visual outcome. However, because macular ischemia was incompletely and inconsistently reported across the included trials, ischemia-based subgroup analysis could not be performed. In addition, a post-hoc qualitative comparison of injection regimens (fixed monthly, PRN after baseline, and loading plus PRN/T&E-like regimens) did not reveal a consistent pattern of differential treatment effects across either BCVA or central retinal thickness outcomes. Given the small number of included trials, any potential effect modification by regimen remains exploratory.

From a mechanistic perspective, both ranibizumab and bevacizumab target vascular endothelial growth factor-A (VEGF-A) [37, 38]. Although differences in molecular structure and pharmacokinetic properties exist, their shared inhibition of VEGF signaling provides a plausible biological basis for comparable short-term anatomical and visual outcomes. Importantly, differences in systemic pharmacokinetic exposure between these agents may further provide context for their systemic pharmacodynamic effects. Ranibizumab has been reported to exhibit lower systemic exposure and reduced systemic VEGF suppression compared with bevacizumab, which provides a biologically plausible context for interpreting differences in systemic safety outcomes [39].

Previous comparative evidence on pharmacological treatments for macular edema secondary to retinal vein occlusion has evolved across multiple methodological layers. Early network meta-analyses were restricted to indirect comparisons among anti-VEGF agents (e.g., Ford et al. [29], Sangroongruangsri et al. [31]), while later studies expanded the analytical framework to include different drug classes such as corticosteroids (Qian et al. [32]), and even broader treatment strategies incorporating laser therapy and combination regimens (Regnier et al. [30], Gao et al. [33]). More recent analyses have further extended this framework by incorporating newer anti-VEGF agents with distinct mechanisms of action, such as faricimab (Chen et al. [34]). While these approaches provide a comprehensive overview of available interventions, they also introduce increasing heterogeneity at the levels of population, pharmacological mechanisms, and treatment strategies, potentially challenging key assumptions such as transitivity and complicating the interpretation of indirect comparisons. Importantly, direct head-to-head evidence comparing ranibizumab and bevacizumab was either absent or only minimally represented in these analyses, and comparative estimates between these two agents were therefore largely driven by indirect evidence. This distinction is particularly relevant given that indirect comparisons rely on assumptions such as transitivity, which may be difficult to fully satisfy across heterogeneous trial settings. In contrast, the present study is positioned at the most internally valid level of evidence by focusing exclusively on direct head-to-head randomized comparisons between ranibizumab and bevacizumab. This approach minimizes heterogeneity related to intervention and comparison structure, and provides a more robust estimate of their comparative efficacy and safety. Therefore, the intended contribution of this study is not to claim that ranibizumab and bevacizumab are previously unstudied treatments, but to provide a focused synthesis of the available direct randomized evidence for a clinically and economically relevant comparison.

Given the absence of evidence demonstrating superiority of ranibizumab over bevacizumab in visual or anatomical outcomes at 6 months, these findings have potential implications for cost-conscious clinical decision-making. In many healthcare systems, bevacizumab is substantially less expensive than ranibizumab (bevacizumab $4,100/year, ranibizumab $18,600/year) [27, 28], and treatment burden in RVO often requires repeated intravitreal injections [40]. When efficacy appears comparable and safety signals are not clearly differentiated, drug cost and accessibility may reasonably influence therapeutic choice, particularly in resource-limited settings. However, long-term comparative data remain limited, and decisions should continue to be individualized based on patient characteristics, regulatory considerations, and institutional policies.

The generalizability of these findings should also be considered in the context of evolving anti-VEGF therapies. This analysis focused specifically on ranibizumab and bevacizumab and should not be extrapolated directly to newer or alternative agents such as aflibercept, faricimab, or conbercept, which may differ in molecular targets, durability, treatment intervals, and regulatory status [41–43]. Nevertheless, ranibizumab and bevacizumab remain clinically relevant in many healthcare settings, particularly where treatment cost, accessibility, and off-label drug availability influence clinical decision-making. Future direct comparative studies in contemporary treatment settings are needed to clarify how these findings relate to broader anti-VEGF treatment choices.

This study has several strengths. By restricting inclusion to randomized controlled trials with direct head-to-head comparisons between ranibizumab and bevacizumab, indirect comparison bias was minimized. Methodological rigor was further enhanced through outcome-level risk-of-bias assessment using the Cochrane RoB 2 tool with predefined decision rules, prespecified statistical strategies tailored to outcome types, and systematic evaluation of evidence certainty using the GRADE framework.

However, several limitations should be acknowledged. First, only four randomized controlled trials were included, and some outcomes were informed by a small number of studies, limiting statistical power and the ability to explore clinical heterogeneity. Although baseline characteristics were generally balanced between treatment arms within individual trials, the included studies differed in RVO subtype, disease duration, baseline anatomical severity, prior treatment status, and the reporting or inclusion of macular ischemia. These factors may influence visual and anatomical responses in RVO-ME. Therefore, the pooled estimates should be interpreted as the average relative treatment effect within the available direct randomized evidence, rather than as a universally applicable estimate across all RVO-ME phenotypes. Second, the main efficacy conclusions were based largely on 6-month outcomes, because most included randomized comparisons reported extractable visual and anatomical data at this time point. Therefore, the present findings should primarily be interpreted as evidence for short-term comparative efficacy, and the extent to which these results can be extrapolated to long-term treatment durability, injection burden, recurrence of macular edema, and cumulative safety remains uncertain. Third, systemic adverse events were rare, resulting in substantial imprecision and uncertainty in safety estimates.

Future research should prioritize long-term randomized comparisons to assess durability of treatment effects and cumulative safety. In addition, individual patient data meta-analyses may help explore potential effect modifiers, such as RVO subtype or baseline visual acuity. Given the rarity of systemic adverse events, large-scale observational or registry-based studies may also provide complementary evidence to improve safety assessment.

## Conclusion

In summary, current randomized evidence does not demonstrate a statistically significant difference between ranibizumab and bevacizumab in short-term visual or anatomical outcomes for RVO-ME. Available safety data do not demonstrate a statistically significant difference between the two agents, although estimates for rare systemic events remain uncertain because of limited statistical power. Overall, the available evidence has not demonstrated a clinically important difference between the two agents in short-term outcomes; however, uncertainty remains due to the limited number of trials, rare safety events, and lack of long-term comparative data.

## Electronic supplementary material

Below is the link to the electronic supplementary material.

Supplementary material 1

Supplementary material 2

Supplementary material 3

Supplementary material 4

Supplementary material 5

## Data Availability

All data generated or analyzed during this study are included in this published article and its supplementary information files. Additional datasets are available from the corresponding author upon reasonable request.

## References

[1] I M, H A. Retinal vein occlusion review. Asia-Pac J Ophthalmol (Phila). 2017;7(1). 10.22608/APO.2017163442. 10.22608/APO.2017442 29280368

[2] Rogers S, McIntosh RL, Cheung N, Lim L, Wang JJ, Mitchell P, et al. The prevalence of retinal vein occlusion: pooled data from population studies from the United States, Europe, Asia, and Australia. Ophthalmology. 2010;117(2):313. 10.1016/j.ophtha.2009.07.017. 20022117 10.1016/j.ophtha.2009.07.017 PMC2945292

[3] de Carlo TE, Romano A, Waheed NK, Duker JS. A review of optical coherence tomography angiography (OCTA). Int J Retin Vitr. 2015;1(1):5. 10.1186/s40942-015-0005-8. 10.1186/s40942-015-0005-8 PMC5066513 27847598

[4] Song P, Xu Y, Zha M, Zhang Y, Rudan I. Global epidemiology of retinal vein occlusion: a systematic review and meta-analysis of prevalence, incidence, and risk factors. J Global Health. 2019;9(1):010427. 10.7189/jogh.09.010427. 10.7189/jogh.09.010427 PMC6513508 31131101

[5] Schmidt-Erfurth U, Garcia-Arumi J, Gerendas BS, Midena E, Sivaprasad S, Tadayoni R, et al. Guidelines for the management of retinal vein occlusion by the European society of retina specialists (EURETINA). Ophthalmologica. 2019;242(3):123–62. 10.1159/000502041. 31412332 10.1159/000502041

[6] Candan Ö, Orman G, Ünlü N, Üney G, Burcu A. Prevalence of retinal vascular diseases in a tertiary care hospital in Türkiye: a hospital-based epidemiologic study. Turk J Ophthalmol. 2025;55(1):16–23. 10.4274/tjo.galenos.2025.88262. 40013490 10.4274/tjo.galenos.2025.88262 PMC11866982

[7] Dinah C, et al. Response to: ‘comment on: ‘treatment patterns and long-term outcomes in anti-VEGF-treated macular oedema secondary to retinal vein occlusion: a retrospective observational study”. Eye. Nat Publishing Group. 2026;40(1):107–16. 10.1038/s41433-026-04645-4. 10.1038/s41433-026-04645-4 PMC13415807 42321515

[8] Opremcak EM, Bruce RA, Lomeo MD, Ridenour CD, Letson AD, Rehmar AJ. Radial optic neurotomy for central retinal vein occlusion: a retrospective pilot study of 11 consecutive cases. Retina. 2001;21(5):408–15. 10.1097/00006982-200110000-00002. 11642369 10.1097/00006982-200110000-00002

[9] Liang X-L, Chen H-Y, Huang Y-S, Au Eong K-GA, Liu X, Yan H, et al. Pars plana vitrectomy and internal limiting membrane peeling for macular oedema secondary to retinal vein occlusion: a pilot study. Ann Acad Med Singap. 2007;36(4):285–92. 10.47102/annals-acadmedsg.V36N4p293. 17483861

[10] Opremcak ME, Bruce RA. Surgical decompression of branch retinal vein occlusion via arteriovenous crossing sheathotomy: a prospective review of 15 cases. Retina. 1999;19(1):1–5. 10.1097/00006982-199901000-00001. 10048366 10.1097/00006982-199901000-00001

[11] Chung EJ, Lee H, Koh HJ. Arteriovenous crossing sheathotomy versus intravitreal triamcinolone acetonide injection for treatment of macular edema associated with branch retinal vein occlusion. Graefes Arch Clin Exp Ophthalmol. 2008;246(7):967–74. 10.1007/s00417-008-0830-7. 18425522 10.1007/s00417-008-0830-7

[12] Branch Vein Occlusion Study Group T. Argon laser photocoagulation for macular edema in branch vein occlusion. The branch vein occlusion study group. Am J Ophthalmol. 1984;98(3):271–82. 10.1016/0002-9394(84)90316-7. 6383055 10.1016/0002-9394(84)90316-7

[13] Campochiaro PA, Hafiz G, Mir TA, Scott AW, Solomon S, Zimmer-Galler I, et al. Scatter photocoagulation does not reduce macular edema or treatment burden in patients with retinal vein occlusion. Ophthalmology. 2015;122(7):1426–37. 10.1016/j.ophtha.2015.04.006. 25972260 10.1016/j.ophtha.2015.04.006 PMC10020833

[14] Frederiksen KH, Vestergaard JP, Pedersen FN, Vergmann AS, Sørensen TL, Laugesen CS, et al. Navigated laser and aflibercept versus aflibercept monotherapy in treatment-naïve branch retinal vein occlusion: a 12-month randomized trial. Acta Ophthalmologica. 2022;100(7):e1503. 10.1111/aos.15182. 10.1111/aos.15182 PMC9790367 35611568

[15] Romano F, Lamanna F, Gabrielle PH, Teo KYC, Battaglia Parodi M, Iacono P, et al. Update on retinal vein occlusion. Asia-Pac J Ophthalmol. 2023;12(2):196–210. 10.1097/APO.0000000000000598. 10.1097/APO.0000000000000598 36912792

[16] Nicholson L, Talks SJ, Amoaku W, Talks K, Sivaprasad S. Retinal vein occlusion (RVO) guideline: executive summary. Eye. Nat Publishing Group. 2022;36(5):909–12. 10.1038/s41433-022-02007-4. 10.1038/s41433-022-02007-4 PMC9046155 35301458

[17] Haller JA, Bandello F, Belfort R, Blumenkranz MS, Gillies M, Heier J, et al. Randomized, sham-controlled trial of dexamethasone intravitreal implant in patients with macular edema due to retinal vein occlusion. Ophthalmology. 2010;117(6):1134–46.e3. 10.1016/j.ophtha.2010.03.032. 20417567 10.1016/j.ophtha.2010.03.032

[18] Karataş G, Uzundede T, Aday Ö, Özoğuz AM, Karataş ME, Kabakcı AK, et al. Dexamethasone intravitreal implant monotherapy in naive patients with macular edema secondary to retinal vein occlusion: long term follow-up retrospective cohort study. Int J Ophthalmol. 2025;18(5):876–82. 10.18240/ijo.2025.05.13. 40385126 10.18240/ijo.2025.05.13 PMC12043306

[19] Ramezani A, Esfandiari H, Entezari M, Moradian S, Soheilian M, Dehsarvi B, et al. Three intravitreal bevacizumab versus two intravitreal triamcinolone injections in recent onset central retinal vein occlusion. Acta Ophthalmologica. 2014;92(7):e530–9. 10.1111/aos.12317. 10.1111/aos.12317 24373344

[20] Hattenbach L-O, Feltgen N, Bertelmann T, Schmitz-Valckenberg S, Berk H, Eter N, et al. Head-to-head comparison of ranibizumab PRN versus single-dose dexamethasone for branch retinal vein occlusion (COMRADE-B). Acta Ophthalmologica. 2018;96(1):e10–8. 10.1111/aos.13381. 10.1111/aos.13381 28251811

[21] Garay-Aramburu G, Hunt A, Arruabarrena C, Mehta H, Invernizzi A, Gabrielle P-H, et al. Initial response and 12-month outcomes after commencing dexamethasone or vascular endothelial growth factor inhibitors for retinal vein occlusion in the FRB registry. Sci Rep Nat Publishing Group. 2024;14(1):6122. 10.1038/s41598-024-56581-6. 10.1038/s41598-024-56581-6 PMC10937938 38480837

[22] Vader MJC, Schauwvlieghe S, Verbraak FD, Dijkman G, Hooymans JMM, Los LI, et al. Comparing the efficacy of Bevacizumab and Ranibizumab in patients with retinal vein occlusion: the Bevacizumab to Ranibizumab in retinal vein occlusions (BRVO) study, a randomized trial. Ophthalmol Retina. 2020;4(6):576–87. 10.1016/j.oret.2019.12.019. 32107188 10.1016/j.oret.2019.12.019

[23] Jumper JM, Dugel PU, Chen S, Blinder KJ, Walt JG. Anti-VEGF treatment of macular edema associated with retinal vein occlusion: patterns of use and effectiveness in clinical practice (ECHO study report 2). OPTH. 2018;12:621–29. 10.2147/OPTH.S163859. 10.2147/OPTH.S163859 PMC5892950 29662298

[24] Yao J, Huang W, Gao L, Liu Y, Zhang Q, He J, et al. Comparative efficacy of anti-vascular endothelial growth factor on diabetic macular edema diagnosed with different patterns of optical coherence tomography: a network meta-analysis. PLoS ONE. Public Lib Sci. 2024;19(6):e0304283. 10.1371/journal.pone.0304283. 10.1371/journal.pone.0304283 PMC11161126 38848379

[25] Parikh R, Ross JS, Sangaralingham LR, Adelman RA, Shah ND, Barkmeier AJ. Trends of Anti-vascular endothelial growth Factor use in ophthalmology among privately insured and Medicare advantage patients. Ophthalmology. 2017;124(3):352–58. 10.1016/j.ophtha.2016.10.036. 27890437 10.1016/j.ophtha.2016.10.036

[26] Singh S, Saxena S, Akduman L, Meyer CH. Off-label use of intravitreal bevacizumab: a global conundrum. Indian J Ophthalmol. 2024;72(5):617–19. 10.4103/IJO.IJO_2166_23. 38661271 10.4103/IJO.IJO_2166_23 PMC11168559

[27] Ross EL, Hutton DW, Stein JD, Bressler NM, Jampol LM, Glassman AR, et al. Cost-effectiveness of aflibercept, Bevacizumab, and ranibizumab for diabetic macular edema treatment: analysis from the diabetic retinopathy clinical research network comparative effectiveness trial. JAMA Ophthalmol. 2016;134(8):888–96. 10.1001/jamaophthalmol.2016.1669. 27280850 10.1001/jamaophthalmol.2016.1669 PMC6648661

[28] van Asten F, Michels CTJ, Hoyng CB, van der Wilt GJ, Klevering BJ, Rovers MM, et al. The cost-effectiveness of bevacizumab, ranibizumab and aflibercept for the treatment of age-related macular degeneration—A cost-effectiveness analysis from a societal perspective. PLoS ONE. 2018;13(5):e0197670. 10.1371/journal.pone.0197670. 10.1371/journal.pone.0197670 PMC5957378 29772018

[29] Ford JA, Shyangdan D, Uthman OA, Lois N, Waugh N. Drug treatment of macular oedema secondary to central retinal vein occlusion: a network meta-analysis. BMJ Open. 2014;4(7):e005292. 10.1136/bmjopen-2014-005292. 10.1136/bmjopen-2014-005292 PMC4120318 25056974

[30] Regnier SA, Larsen M, Bezlyak V, Allen F. Comparative efficacy and safety of approved treatments for macular oedema secondary to branch retinal vein occlusion: a network meta-analysis. BMJ Open. 2015;5(6):e007527. 10.1136/bmjopen-2014-007527. 10.1136/bmjopen-2014-007527 PMC4458587 26048209

[31] Sangroongruangsri S, Ratanapakorn T, Wu O, Anothaisintawee T, Chaikledkaew U. Comparative efficacy of bevacizumab, ranibizumab, and aflibercept for treatment of macular edema secondary to retinal vein occlusion: a systematic review and network meta-analysis. Expert Rev Clin Pharmacol. 2018;11(9):903–16. 10.1080/17512433.2018.1507735. 30071180 10.1080/17512433.2018.1507735

[32] Qian T, Zhao M, Wan Y, Li M, Xu X. Comparison of the efficacy and safety of drug therapies for macular edema secondary to central retinal vein occlusion. BMJ Open. 2018;8(12):e022700. 10.1136/bmjopen-2018-022700. 10.1136/bmjopen-2018-022700 PMC6318534 30593547

[33] Gao S, Zhang Y, Li X, Ge G, Duan J, Lei C, et al. Comparative efficacy of pharmacotherapy for macular edema secondary to retinal vein occlusion: a network meta-analysis. Front Pharmacol. 2021;12:12. 10.3389/fphar.2021.752048. 10.3389/fphar.2021.752048 PMC8692786 34955825

[34] Chen K-Y, Chan H-C, Chan C-M. Effectiveness and safety of anti-vascular endothelial growth factor therapies for macular edema in retinal vein occlusion: a systematic review and network meta-analysis of randomized controlled trials. Surv Ophthalmol. 2025;70(6):1067–89. 10.1016/j.survophthal.2025.05.008. 40419166 10.1016/j.survophthal.2025.05.008

[35] Page MJ, McKenzie JE, Bossuyt PM, Boutron I, Hoffmann TC, Mulrow CD, et al. The PRISMA, 2020 statement: an updated guideline for reporting systematic reviews. Br Med J Publishing Group. 2021. 10.1136/bmj.n71. 10.1136/bmj.n71 PMC8005924 33782057

[36] Sterne JAC, Savović J, Page MJ, Elbers RG, Blencowe NS, Boutron I, et al. RoB 2: a revised tool for assessing risk of bias in randomised trials. BMJ. 2019;366:l4898. 10.1136/bmj.l4898. 10.1136/bmj.l4898 31462531

[37] Funk M, Kriechbaum K, Prager F, Benesch T, Georgopoulos M, Zlabinger GJ, et al. Intraocular concentrations of growth factors and cytokines in retinal vein occlusion and the effect of therapy with bevacizumab. Invest Ophthalmol Vis Sci. 2009;50(3):1025–32. 10.1167/iovs.08-2510. 19060280 10.1167/iovs.08-2510

[38] Arepalli S, Wykoff CC, Abraham JR, Lunasco L, Yu H, Hu M, et al. Longitudinal analysis of aqueous humour cytokine expression and OCT-based imaging biomarkers in retinal vein occlusions treated with anti-vascular endothelial growth factor therapy in the IMAGINE study. Eye (Lond). 2023;37(9):1928–35. 10.1038/s41433-022-02265-2. 36220884 10.1038/s41433-022-02265-2 PMC10275974

[39] Avery RL, Castellarin AA, Steinle NC, Dhoot DS, Pieramici DJ, See R, et al. Systemic pharmacokinetics and pharmacodynamics of intravitreal aflibercept, bevacizumab, and ranibizumab. Retina Philadelphia, Pa. 2017;37(10):1847–58. 10.1097/IAE.0000000000001493. 28106709 10.1097/IAE.0000000000001493 PMC5642319

[40] Brown DM, Campochiaro PA, Singh RP, Li Z, Gray S, Saroj N, et al. Ranibizumab for macular edema following central retinal vein occlusion: six-month primary end point results of a phase III study. Ophthalmol Elsevier. 2010;117(6):1124–33.e1. 10.1016/j.ophtha.2010.02.022. 10.1016/j.ophtha.2010.02.022 20381871

[41] Sun X, Lu X. Profile of conbercept in the treatment of neovascular age-related macular degeneration. DDDT. 2015;9:2311–20. 10.2147/DDDT.S67536. 25960634 10.2147/DDDT.S67536 PMC4410828

[42] Suresh B, Patel P. Aflibercept. In: StatPearls. [Internet]. Treasure Island (FL): StatPearls Publishing; 2026 [cited 2026 May 5]. Available from: http://www.ncbi.nlm.nih.gov/books/NBK582136/.

[43] Khanani AM, Guymer RH, Basu K, Boston H, Heier JS, Korobelnik J-F, et al. Tenaya and Lucerne: rationale and design for the phase 3 clinical trials of Faricimab for neovascular age-related macular degeneration. Ophthalmol Sci. 2021;1(4):100076. 10.1016/j.xops.2021.100076. 36246941 10.1016/j.xops.2021.100076 PMC9559073

