# Supplementary material for: Direct randomized evidence comparing ranibizumab and bevacizumab for macular edema secondary to retinal vein occlusion: a systematic review and meta-analysis
Source: BMC Ophthalmol. 2026 Jul 30;26:449. doi: 10.1186/s12886-026-05146-4 (PMC13421823; doi:10.1186/s12886-026-05146-4)
Supplement: Supplementary file 1 — Supplementary material 1 [file 12886_2026_5146_MOESM1_ESM.docx]

|  | **Embase** |  |
| --- | --- | --- |
| #1 | retinal vein occlusion'/exp OR 'retinal vein occlusion' OR (('retinal'/exp OR retinal) AND ('vein'/exp OR vein) AND ('occlusion'/exp OR occlusion)) OR 'retinal vein occlusion':ab,ti OR 'retinal branch vein occlusion':ab,ti OR 'central retinal vein occlusion':ab,ti OR 'central retinal venous occlusion':ab,ti OR 'branch retinal vein occlusion':ab,ti OR 'retinal vein thrombosis':ab,ti OR brvo:ab,ti OR crvo:ab,ti OR rvo:ab,ti | 16169 |
| #2 | 'macular edema'/exp OR 'macular edema' OR 'macular edema':ab,ti OR 'macular swelling':ab,ti OR 'macular thickening':ab,ti OR 'retinal edema':ab,ti OR 'macular oedema':ab,ti OR 'edema of macula':ab,ti OR me:ab,ti | 122695 |
| #3 | #1 AND #2 | 6396 |
| #4 | 'ranibizumab'/exp OR ranibizumab OR ranibizumab:ab,ti OR lucentis:ab,ti OR byooviz:ab,ti OR ranivisio:ab,ti OR razumab:ab,ti OR 'rhufab v2':ab,ti | 16023 |
| #5 | 'bevacizumab'/exp OR bevacizumab OR 'bevacizumab awwb':ab,ti OR avastin:ab,ti OR mvasi:ab,ti OR 'zirabev'/exp OR zirabev OR 'alymsys'/exp OR alymsys OR vegzelma:ab,ti | 96362 |
| #6 | #3 AND #4 AND #5 | 813 |
| #7 | #6 AND 'randomized controlled trial topic'/de | 101 |

**Table S1** Embase re-run search strategy (June 30, 2026)

**Note**: The Embase search was re-run on June 30, 2026 using corrected OR logic for the RVO abbreviation terms (“brvo”, “crvo”, and “rvo”). No additional eligible randomized controlled trials were identified.
