# Supplementary material for: Direct randomized evidence comparing ranibizumab and bevacizumab for macular edema secondary to retinal vein occlusion: a systematic review and meta-analysis
Source: BMC Ophthalmol. 2026 Jul 30;26:449. doi: 10.1186/s12886-026-05146-4 (PMC13421823; doi:10.1186/s12886-026-05146-4)
Supplement: Supplementary file 2 — Supplementary material 2 [file 12886_2026_5146_MOESM2_ESM.docx]

**Table 1A** Baseline clinical and phenotypic characteristics of included randomized controlled trials

| **Study_ID** | **RVO type** | **RVO subtype distribution** | **Baseline BCVA** | **Baseline macular thickness / OCT measure** | **Disease duration** | **Ischemia/perfusion-related information** | **Prior treatment status** | **Key inclusion/exclusion notes** | **Follow-up duration** |
| --- | --- | --- | --- | --- | --- | --- | --- | --- | --- |
| Narayanan 2015 | BRVO | BRVO only | Ranibizumab: 52.81±14.41 ETDRS letters; Bevacizumab: 56.10±10.01 ETDRS letters | Ranibizumab: CRT 445.65±119.53 μm; Bevacizumab: CRT 491.55±155.09 μm | BRVO-related center-involving macular edema of <9 months duration | Macular ischemia status not explicitly reported; FA was performed at baseline and repeated at 6 months. | Treatment-naive to anti-VEGF in the study eye; excluded previous PRP or macular laser photocoagulation in the study eye, intraocular/periocular corticosteroids within 3 months, and previous vitreoretinal surgery. | Adults ≥18 years; center-involving macular edema due to BRVO; minimum central subfield CRT 250 μm on SD-OCT; BCVA 20/40 to 20/320, equivalent to 73 to 24 ETDRS letters; decreased VA from causes other than BRVO excluded. | 6 months; monthly visits through week 24; baseline injection followed by monthly PRN retreatment |
| Rajagopal 2015 | Mixed RVO | Ranibizumab: BRVO/HRVO , CRVO ; Bevacizumab: BRVO/HRVO, CRVO | Ranibizumab: 0.73±0.45 logMAR.  Bevacizumab: 0.76±0.38 logMAR; | Ranibizumab: CFT 517.2±159.8 μm.  Bevacizumab: CFT 538.0±211.0 μm; | Mean onset of RVO: ranibizumab 1.6±1.7 months; bevacizumab 1.3±1.1 months. | Ischemic RVOs were included; FA was performed at baseline and weeks 12 and 24, but baseline ischemia/perfusion distribution was not reported in this 6-month report. | Excluded intravitreal injections within 12 weeks, prior RVO, PRP within 3 months or anticipated within 4 months, and intraocular surgery within 60 days except uncomplicated cataract surgery. | Age >50 years; RVO diagnosed within past 9 months; BCVA 20/40 to 20/320; CFT >250 μm on OCT; included regardless of relative afferent pupillary defect; excluded diabetic retinopathy/DME, uncontrolled glaucoma, recent MI/stroke, pregnancy/lactation. | 12 months total; 6 monthly mandatory injections during first 6 months; 6-month findings reported as primary analysis |
| Vader 2020 | Mixed RVO | Ranibizumab: BRVO, CRVO, hemi-CRVO.  Bevacizumab: BRVO, CRVO, hemi-CRVO; | Ranibizumab: 59.0±16.7 ETDRS letters, Bevacizumab: 60.3±14.8 ETDRS letters; | Ranibizumab: CAT 615.2±217.3 μm, Bevacizumab: CAT 602.3±201.2 μm; | NR; eligibility required vision loss due to ME secondary to RVO, but disease duration or time from diagnosis was not reported. | Baseline macular ischemia/perfusion status was not reported in the original baseline characteristics table; eligibility assessment included FA, and FA was performed at screening and 6 months. | Prior RVO in study eye, ranibizumab vs bevacizumab: 17/138 vs 19/139; prior anti-VEGF: 6/138 vs 4/139; prior photocoagulation: 3/138 vs 2/139. | Adults >18 years; vision loss due to ME secondary to BRVO, CRVO, or hemi-CRVO; central area thickness >275 μm on OCT; BCVA >24 and <79 ETDRS letters; diagnosis confirmed by the Belfast Reading Centre. | 6 months; monthly dosing interval; 6 monthly injections |
| Hykin 2021 | CRVO | CRVO only | Ranibizumab: 53.6±15.1 ETDRS letters; Bevacizumab: 54.4±14.2 ETDRS letters | Ranibizumab: CST 731.3±227.6 μm; Bevacizumab: CST 676.1±207.0 μm | Median CRVO duration: ranibizumab 0.9 months (IQR 0.5–1.8); bevacizumab 0.9 months (IQR 0.4–1.7). Duration <3 months: 134/155 vs 138/154. | Baseline CRVO ischaemic status: non-ischaemic/ischaemic, ranibizumab 137/17; bevacizumab 134/20. | Previous treatment in trial eye, ranibizumab vs bevacizumab: nil 148 vs 149; prior anti-VEGF therapy 6 vs 5. | Adults ≥18 years; centre-involving MO due to CRVO; CRVO ≤12 months; BCVA 19–78 ETDRS letters; OCT CST >320 μm or equivalent. Excluded active neovascularisation/NVG/vitreous haemorrhage, uncontrolled glaucoma, recent corticosteroid or anti-VEGF treatment beyond allowed limits, uncontrolled blood pressure, and recent MI/stroke/TIA/acute coronary event. | 100 weeks; mandated injections at baseline and weeks 4, 8, and 12, followed by protocol-defined PRN treatment with possible extension of visit interval from 4 to 8 weeks. |
| HRVO=Hemiretinal Vein Occlusion; CFT=Central Foveal Thickness; CAT=Central Area Thickness; ETDRS=Early Treatment Diabetic Retinopathy Study; FA=Fluorescein Angiography; IQR=Interquartile Range; ME=Macular Edema; MI=Myocardial Infarction; MO=Macular Oedema; NR=Not Reported; NVG=Neovascular Glaucoma; PRP=Panretinal Photocoagulation; TIA=Transient Ischemic Attack. | | | | | | | | | |
