# Supplementary material for: Direct randomized evidence comparing ranibizumab and bevacizumab for macular edema secondary to retinal vein occlusion: a systematic review and meta-analysis
Source: BMC Ophthalmol. 2026 Jul 30;26:449. doi: 10.1186/s12886-026-05146-4 (PMC13421823; doi:10.1186/s12886-026-05146-4)
Supplement: Supplementary file 3 — Supplementary material 3 [file 12886_2026_5146_MOESM3_ESM.docx]

**Table S** Search Query

| **Search number** | **Search Query** | **Results** |
| --- | --- | --- |
|  | **Cochrane** |  |
| #1 | MeSH descriptor: [Retinal Vein Occlusion] explode all trees | 474 |
| #2 | (retinal vein occlusion):ti,ab,kw OR (Retinal Vein Occlusion):ti,ab,kw OR (Retinal branch vein occlusion):ti,ab,kw OR (Central Retinal Vein Occlusion):ti,ab,kw OR (Central retinal venous occlusion):ti,ab,kw | 1079 |
| #3 | (Branch Retinal Vein Occlusion):ti,ab,kw OR (Retinal Vein Thrombosis):ti,ab,kw OR (BRVO):ti,ab,kw OR (CRVO):ti,ab,kw OR (RVO):ti,ab,kw | 847 |
| #4 | #1 or #2 or #3 | 1157 |
| #5 | MeSH descriptor: [Macular Edema] explode all trees | 1593 |
| #6 | (Macular Edema):ti,ab,kw OR (Macular swelling):ti,ab,kw OR (Macular thickening):ti,ab,kw OR (Retinal edema):ti,ab,kw OR (Macular oedema):ti,ab,kw | 4507 |
| #7 | (Edema of macula):ti,ab,kw OR (ME):ti,ab,kw | 5474 |
| #8 | #5 or #6 or #7 | 9000 |
| #9 | #4 and #8 | 776 |
| #10 | MeSH descriptor: [Ranibizumab] explode all trees | 1229 |
| #11 | (Ranibizumab):ti,ab,kw OR (Lucentis):ti,ab,kw OR (Byooviz):ti,ab,kw OR (Ranivisio):ti,ab,kw OR (Razumab):ti,ab,kw | 2435 |
| #12 | (RhuFab V2):ti,ab,kw | 20 |
| #13 | #10 or #11 or #12 | 2440 |
| #14 | MeSH descriptor: [Bevacizumab] explode all trees | 3162 |
| #15 | (Bevacizumab):ti,ab,kw OR (Bevacizumab awwb):ti,ab,kw OR (Avastin):ti,ab,kw OR (Mvasi):ti,ab,kw OR (Zirabev):ti,ab,kw | 8576 |
| #16 | (Alymsys):ti,ab,kw OR (Vegzelma):ti,ab,kw | 2 |
| #17 | #14 or #15 or #16 | 8576 |
| #18 | #9 and #13 and #17 | 51 |
|  | **Embase** |  |
| #1 | ('retinal vein occlusion'/exp OR 'retinal vein occlusion' OR (('retinal'/exp OR retinal) AND ('vein'/exp OR vein) AND ('occlusion'/exp OR occlusion)) OR 'retinal vein occlusion':ab,ti OR 'retinal branch vein occlusion':ab,ti OR 'central retinal vein occlusion':ab,ti OR 'central retinal venous occlusion':ab,ti OR 'branch retinal vein occlusion':ab,ti OR 'retinal vein thrombosis':ab,ti OR brvo:ab,ti) AND crvo:ab,ti AND rvo:ab,ti | 556 |
| #2 | 'macular edema'/exp OR 'macular edema' OR 'macular edema':ab,ti OR 'macular swelling':ab,ti OR 'macular thickening':ab,ti OR 'retinal edema':ab,ti OR 'macular oedema':ab,ti OR 'edema of macula':ab,ti OR me:ab,ti | 119702 |
| #3 | #1 AND #2 | 310 |
| #4 | 'ranibizumab'/exp OR ranibizumab OR ranibizumab:ab,ti OR lucentis:ab,ti OR byooviz:ab,ti OR ranivisio:ab,ti OR razumab:ab,ti OR 'rhufab v2':ab,ti | 15705 |
| #5 | #3 AND #4 | 121 |
|  | **Pubmed** |  |
| #1 | "retinal vein occlusion"[MeSH Terms] OR "retinal vein occlusion"[Title/Abstract] OR "retinal branch vein occlusion"[Title/Abstract] OR "central retinal vein occlusion"[Title/Abstract] OR "central retinal venous occlusion"[Title/Abstract] OR "branch retinal vein occlusion"[Title/Abstract] OR "retinal vein thrombosis"[Title/Abstract] OR "BRVO"[Title/Abstract] OR "CRVO"[Title/Abstract] OR "RVO"[Title/Abstract] | 8,476 |
| #2 | "macular edema"[MeSH Terms] OR "macular edema"[Title/Abstract] OR "macular swelling"[Title/Abstract] OR "macular thickening"[Title/Abstract] OR "retinal edema"[Title/Abstract] OR "macular oedema"[Title/Abstract] OR (("Edema"[MeSH Terms] OR "Edema"[All Fields] OR "edemas"[All Fields] OR "oedemas"[All Fields] OR "oedema"[All Fields]) AND "of macula"[Title/Abstract]) OR "ME"[Title/Abstract] | 81,750 |
| #3 | "Ranibizumab"[MeSH Terms] OR "Ranibizumab"[Title/Abstract] OR "Lucentis"[Title/Abstract] OR "Byooviz"[Title/Abstract] OR "Ranivisio"[Title/Abstract] OR "Razumab"[Title/Abstract] OR "rhufab v2"[Title/Abstract] | 7,396 |
| #4 | "Bevacizumab"[MeSH Terms] OR "Bevacizumab"[Title/Abstract] OR "bevacizumab awwb"[Title/Abstract] OR "Avastin"[Title/Abstract] OR "Mvasi"[Title/Abstract] OR "Zirabev"[Title/Abstract] OR "Alymsys"[Title/Abstract] OR "Vegzelma"[Title/Abstract] | 26,945 |
| #5 | ("retinal vein occlusion"[MeSH Terms] OR "retinal vein occlusion"[Title/Abstract] OR "retinal branch vein occlusion"[Title/Abstract] OR "central retinal vein occlusion"[Title/Abstract] OR "central retinal venous occlusion"[Title/Abstract] OR "branch retinal vein occlusion"[Title/Abstract] OR "retinal vein thrombosis"[Title/Abstract] OR "BRVO"[Title/Abstract] OR "CRVO"[Title/Abstract] OR "RVO"[Title/Abstract]) AND ("macular edema"[MeSH Terms] OR "macular edema"[Title/Abstract] OR "macular swelling"[Title/Abstract] OR "macular thickening"[Title/Abstract] OR "retinal edema"[Title/Abstract] OR "macular oedema"[Title/Abstract] OR (("Edema"[MeSH Terms] OR "Edema"[All Fields] OR "edemas"[All Fields] OR "oedemas"[All Fields] OR "oedema"[All Fields]) AND "of macula"[Title/Abstract]) OR "ME"[Title/Abstract]) AND ("Ranibizumab"[MeSH Terms] OR "Ranibizumab"[Title/Abstract] OR "Lucentis"[Title/Abstract] OR "Byooviz"[Title/Abstract] OR "Ranivisio"[Title/Abstract] OR "Razumab"[Title/Abstract] OR "rhufab v2"[Title/Abstract]) | 699 |
| #6 | (("retinal vein occlusion"[MeSH Terms] OR "retinal vein occlusion"[Title/Abstract] OR "retinal branch vein occlusion"[Title/Abstract] OR "central retinal vein occlusion"[Title/Abstract] OR "central retinal venous occlusion"[Title/Abstract] OR "branch retinal vein occlusion"[Title/Abstract] OR "retinal vein thrombosis"[Title/Abstract] OR "BRVO"[Title/Abstract] OR "CRVO"[Title/Abstract] OR "RVO"[Title/Abstract]) AND ("macular edema"[MeSH Terms] OR "macular edema"[Title/Abstract] OR "macular swelling"[Title/Abstract] OR "macular thickening"[Title/Abstract] OR "retinal edema"[Title/Abstract] OR "macular oedema"[Title/Abstract] OR (("Edema"[MeSH Terms] OR "Edema"[All Fields] OR "edemas"[All Fields] OR "oedemas"[All Fields] OR "oedema"[All Fields]) AND "of macula"[Title/Abstract]) OR "ME"[Title/Abstract]) AND ("Ranibizumab"[MeSH Terms] OR "Ranibizumab"[Title/Abstract] OR "Lucentis"[Title/Abstract] OR "Byooviz"[Title/Abstract] OR "Ranivisio"[Title/Abstract] OR "Razumab"[Title/Abstract] OR "rhufab v2"[Title/Abstract])) AND (randomizedcontrolledtrial[Filter]) | 85 |
|  | **Wos** |  |
| #1 | (((((((((ALL=(Retinal Vein Occlusion)) OR ALL=(Retinal Vein Occlusion)) OR ALL=(Retinal branch vein occlusion)) OR ALL=(Central Retinal Vein Occlusion)) OR ALL=(Central retinal venous occlusion)) OR ALL=(Branch Retinal Vein Occlusion)) OR ALL=(Retinal Vein Thrombosis)) OR ALL=(BRVO)) OR ALL=(CRVO)) OR ALL=(RVO) | 24671 |
| #2 | (((((((ALL=(Macular Edema)) OR ALL=(Macular Edema)) OR ALL=(Macular swelling)) OR ALL=(Macular thickening)) OR ALL=(Retinal edema)) OR ALL=(Macular oedema)) OR ALL=(Edema of macula)) OR ALL=(ME) | 944674 |
| #3 | ((((((ALL=(Ranibizumab)) OR ALL=(Ranibizumab)) OR ALL=(Lucentis)) OR ALL=(Byooviz)) OR ALL=(Ranivisio)) OR ALL=(Razumab)) OR ALL=(RhuFab V2) | 12338 |
| #4 | #1 AND #2 | 4252 |
| #5 | (((((((ALL=(Bevacizumab)) OR ALL=(Bevacizumab)) OR ALL=(Bevacizumab awwb)) OR ALL=(Avastin)) OR ALL=(Mvasi)) OR ALL=(Zirabev)) OR ALL=(Alymsys)) OR ALL=(Vegzelma) | 47377 |
| #6 | #3 AND #4 AND #5 | 609 |
| #7 | #3 AND #4 AND #5 and Article (Document Types) | 486 |
