# Supplementary figures and images for: Direct randomized evidence comparing ranibizumab and bevacizumab for macular edema secondary to retinal vein occlusion: a systematic review and meta-analysis
Source: BMC Ophthalmol. 2026 Jul 30;26:449. doi: 10.1186/s12886-026-05146-4 (PMC13421823; doi:10.1186/s12886-026-05146-4)

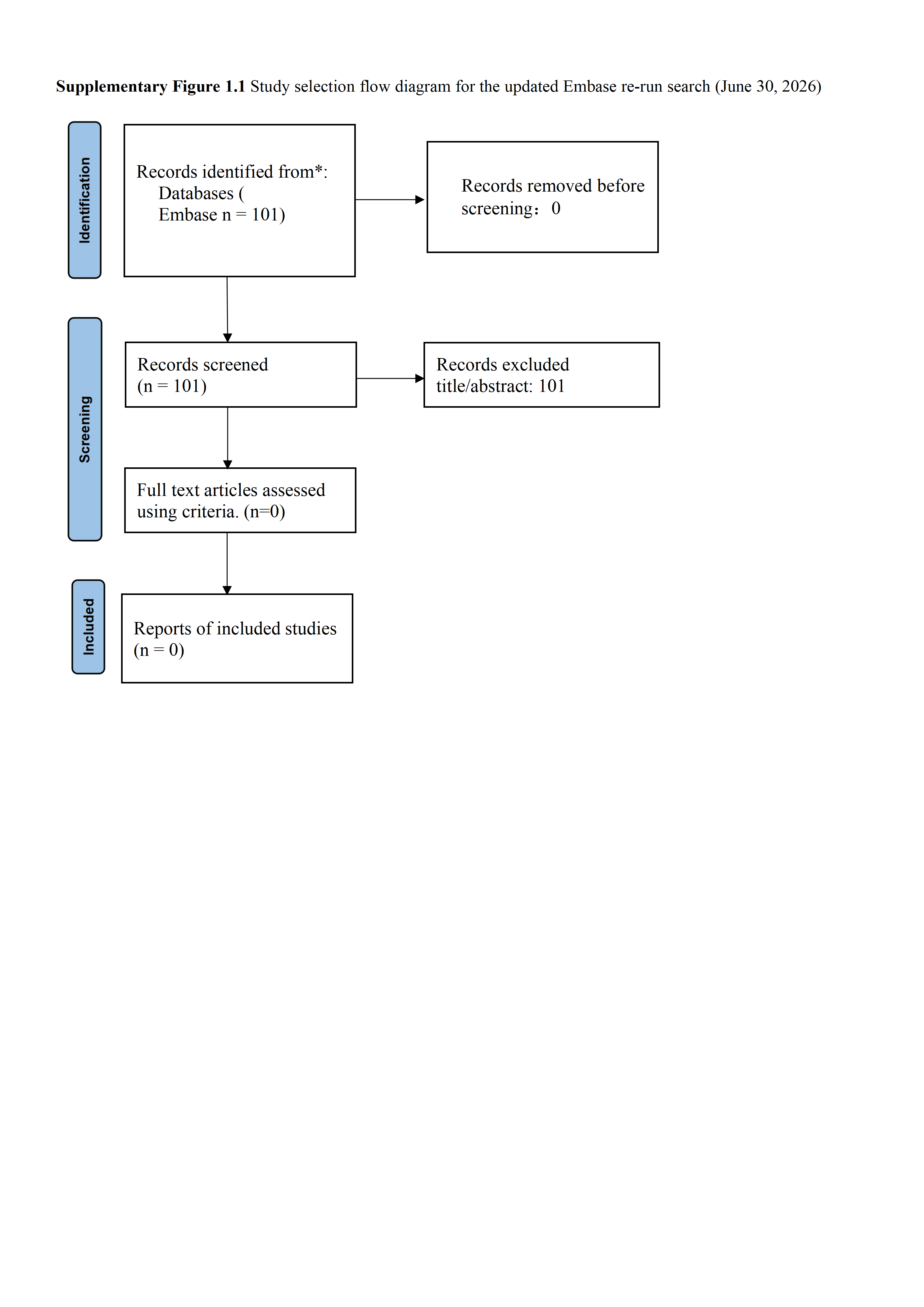

Supplement: Supplementary file 4 — Supplementary material 4 [file 12886_2026_5146_MOESM4_ESM.tiff]
